# Cortical non-aneurysmal subarachnoid hemorrhage post-carotid endarterectomy: a case report and literature review

**DOI:** 10.1186/2193-1801-2-571

**Published:** 2013-10-29

**Authors:** Gopiga Thanabalasundaram, Silvia Hernández-Durán, Thabele Leslie-Mazwi, Christopher S Ogilvy

**Affiliations:** Neurosurgical Service, Harvard Medical School, Massachussetts General Hospital, 55 Fruit Street, Wang, 745 Boston, MA USA; Neuroendovascular Service, Massachusetts General Hospital, 55 Fruit Street, GRB 2-241, Boston, MA 02114 USA; University College London Medical School, Gower Street, London, WC1E 6BT UK; Universidad de Costa Rica, Ciudad Universitaria Rodrigo Facio, San Pedro de Montes de Oca, San José, Costa Rica

**Keywords:** Non-aneurysmal subarachnoid hemorrhage, Carotid endarterectomy, Carotid stenosis, Reperfusion injury

## Abstract

Cerebral hyperperfusion syndrome is a well-recognized and potentially fatal complication of carotid revascularization. However, the occurrence of non-aneurysmal subarachnoid hemorrhage as a manifestation of cerebral hyperperfusion syndrome post-carotid endarterectomy is uncommon. We report a case of a patient who presented with headache following carotid endarterectomy for a critically occluded common carotid artery. This progressed to deteriorating consciousness and seizures. Investigations revealed a left cortical non-aneurysmal subarachnoid hemorrhage. Non-aneurysmal subarachnoid hemorrhage is a rare post-operative complication of carotid endarterectomy. Immediate management with aggressive blood pressure control is key to prevent permanent neurological deficits.

Cerebral hyperperfusion syndrome (CHS) after carotid revascularization procedures is an uncommon and potentially fatal complication. Pathophysiologically it is attributed to impaired autoregulatory mechanisms and results in disruption of cerebral hemodynamics with increased regional cerebral blood flow (Cardiol Rev 20:84–89, 2012; J Vasc Surg 49:1060–1068, 2009). The condition is characterized by throbbing ipsilateral frontotemporal or periorbital headache. Other symptoms include vomiting, confusion, macular edema, focal motor seizures with frequent secondary generalization, focal neurological deficits, and intraparenchymal or subarachnoid hemorrhage (SAH) (Lancet Neurol 4:877–888, 2005). The incidence of CHS varies from 0.2% to 18.9% after carotid endarterectomy (CEA), with a typical reported incidence of less than 3% in larger studies (Cardiol Rev 20:84–89, 2012; Neurosurg 107:1130–1136, 2007). Uncontrolled hypertension, an arterially isolated cerebral hemisphere, and contralateral carotid occlusion are the main risk factors (Lancet Neurol 4:877–888, 2005; J Neurol Neurosurg Psychiatry 83:543–550, 2012). We present a case of non-aneurysmal SAH after CEA, with focus on its presentation, risk factors, and management.

## Case presentation

A 66-year old male presented with severe headache and tonic-clonic seizures on the 6th post-operative day after left CEA surgery at an outside facility. Past medical history included hypertension, hypercholesterolemia, bilateral carotid stenosis and smoking (40 pack-years). At time of left CEA the 99% occluded left carotid artery was revascularized without any apparent complications. Consequently, he was discharged on the 1st post-operative day with no neurological deficits on aspirin, statin and antihypertensives, though his blood pressure proved difficult to control. On the 6th post-operative day, he developed lethargy and drowsiness, followed by sudden onset of the worst headache of his life. Symptoms progressed rapidly to tonic-clonic movements with loss of consciousness. He was emergently intubated by paramedics, and taken to his local medical center. On admission, his blood pressure of 230/170 mmHg, was treated with nicardipine, and he was loaded with levetiracetam. Non-contrast head CT (NCCT) evinced SAH in the left Sylvian fissure, prompting referral to our center for further management.

**Figure 1:**
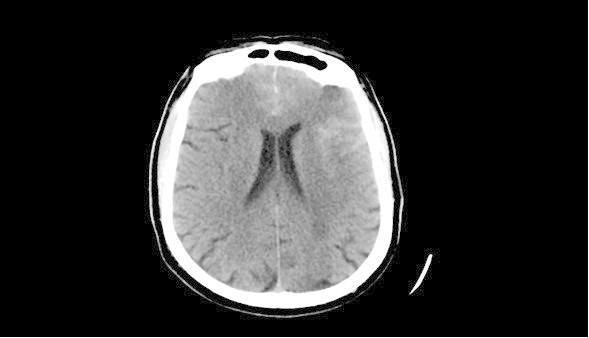
**The CT shows widespread hyperdense subarachnoid hemorrhage, within the left frontal sulci and left Sylvian fissure.**

**Figure 2:**
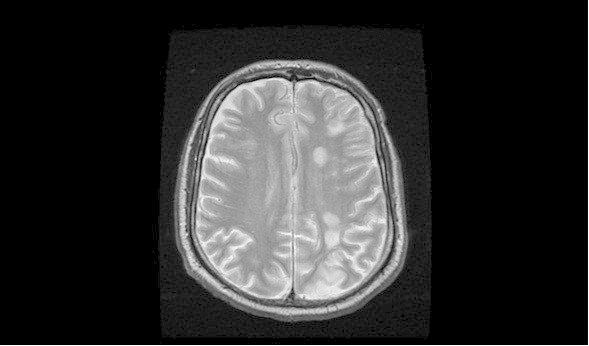
**This axial T2-weighted MRI shows widespread bilateral sulcal FLAIR hyperintensity, which is consistent with SAH.**

Upon admission at our center, examination demonstrated moderate right hemiplegia. Six units of platelets were transfused, and his aspirin was held in order to manage the patient’s bleeding and counteract the anti-aggregation treatment he had. He was started on nimodipine for vasospasm prophylaxis, while continuing on nicardipine for blood pressure control, and levetiracetam for seizure prevention. NCCT confirmed left cortical SAH (see Figure 1), most pronounced in the left frontal and temporal lobe sulci, while CTA showed hypervascularity on the left, but was negative for aneurysm or vascular malformations. His left internal carotid artery was patent, while the right one showed known critical stenosis at its bifurcation. MR perfusion exhibited mild hyperperfusion in the left MCA territory, while the right MCA territory showed hypoperfusion, consistent with the near occlusion of the right ICA (Figure 2). Furthermore, several cortical FLAIR and T2 hyperintensities were observed, that likely reflected sequeale of the recent seizure activity.

Blood pressure control in the Neurosciences Intensive Care unit over the course of several days produced improvement and the patient was discharged to a rehabilitation hospital, able to walk, with mild confusion.

## Discussion

In this report, we describe a case of isolated left-sided SAH 6 days after ipsilateral CEA surgery. No evidence of intraparenchymal hemorrhage or other explanation for the SAH was found. SAH as a manifestation of CHS after CEA is an extremely rare complication; Ogasawara et al. report a 0% incidence of SAH in the CEA (n = 1596) subgroup of their retrospective review of 4494 patients (Ogasawara et al. 2007), whereas McDonald et al. report it as being the least common form of CHS in 203 post-CEA patients (McDonald et al. 2011). To our knowledge, there are only 2 previous cases that report this phenomenon (Bodenant et al. 2010); (Dalton 1992).

In 1992 SAH was described in a 62-year old Caucasian woman 5 days post-CEA surgery with no neurological deficits (Dalton 1992). In 2010 a 74-year old male who presented with left-sided hemiplegia, left visual and sensory neglect, fluctuant altered consciousness and partial motor seizure 9 days following a right CEA was reported (Bodenant et al. 2010). Neuroimaging confirmed a frontal SAH but was negative for aneurysms and vascular malformations. In contrast to our patient, that patient developed vasospasm in the middle cerebral artery. In both these cases, as in our patient, the likely source of the hemorrhage was rupture of small dilated, cortical vessels experiencing elevated flow and pressures following restoration of normal ICA flow.

Post-operative hypertension has been identified as one of the most important risk factors predisposing to CHS in post-CEA patients (Lieb et al. 2012; Ogasawara et al. 2007; van Mook et al. 2005), and Bouri et al. report reduced odds for intracranial hemorrhage when systolic blood pressure is maintained at <140 – 160 mmHg post-operatively (Bouri et al. 2011). Our patient was difficult to maintain normotensive in the post-operative period, and presented with blood pressures of 230/170 mmHg, which likely contributed to development of CHS and the subsequent SAH. A recent study reinforces the importance of treating hypertension to minimize neurological deficits (Wellman & Koide 2013), supporting the strict blood pressure control the patient underwent.

An isolated cerebral hemisphere, whose perfusion is dependent solely upon the ipsilateral carotid artery, is also a risk factor for CHS (Moulakakis et al. 2009; Moulakakis et al. 2012). While our patient did not have an incomplete Circle of Willis, he had bilateral carotid stenosis, and his CTA showed severe right ICA narrowing, which has been associated with an increased likelihood of developing CHS (Pyysalo et al. 2011), likely through disruption of auto-regulatory mechanisms. However, on the other hand, a recent paper earlier this year has found that the degree of contralateral stenosis was not significant in the development in cerebral hyper-perfusion. These results indicate that further research has to be done in the field of contralateral stenosis (Maas et al. 2013).

The risk of significant vasospasm developing from a cortical SAH in this clinical context is minimal, and was not a significant management concern in this case. If concern is higher, due to a significant hemorrhagic component in the basal cisterns, consideration may be given to the use of nimodipine, and longer inpatient monitoring, with cerebrospinal fluid drainage as indicated. Anticonvulsants (used to treat his presumed seizures at onset) are important to ensure adequate seizure control because the spikes in systolic blood pressure during seizure activity can further worsen the established CHS (Connolly et al. 2012).

This case demonstrates the importance of aggressively controlling hypertension in patients after their CEA surgery. The presence of one or more risk factors for CHS demands particular vigilance and outpatient blood pressure monitoring on discharge. The case also highlights a rare presentation of CHS. Appropriate aggressive management of this complication through blood pressure reduction, seizure control and antispasmodic therapy (if indicated) decrease the risk of mortality and neurological deficits (Pyysalo et al. 2011).

### Consent

Informed consent was obtained from the patient for the publication of this report and any accompanying images.
